# Silencing of CD47 and SIRPα by Polypurine reverse Hoogsteen hairpins to promote MCF-7 breast cancer cells death by PMA-differentiated THP-1 cells

**DOI:** 10.1186/s12865-016-0170-z

**Published:** 2016-09-26

**Authors:** Gizem Bener, Alex J. Félix, Cristina Sánchez de Diego, Isabel Pascual Fabregat, Carlos J. Ciudad, Véronique Noé

**Affiliations:** Department of Biochemistry and Molecular Biology, School of Pharmacy, University of Barcelona, IN2UB, Barcelona, Spain

**Keywords:** Immunotherapy, PPRH, CD47, SIRPα, Macrophage

## Abstract

**Background:**

In the context of tumor immunology, tumor cells have been shown to overexpress CD47, an anti-phagocytic signal directed to macrophages to escape from phagocytosis by interacting with Signal Regulatory Protein α SIRPα.

In the present work, we designed Polypurine reverse Hoogsteen hairpins, PPRHs, to silence the expression of CD47 in tumor cells and SIRPα in macrophages with the aim to eliminate tumor cells by macrophages in co-culture experiments.

**Methods:**

THP-1 cells were differentiated to macrophages with PMA. The mRNA levels of differentiation markers CD14 and Mcl-1 mRNA and pro-inflammatory cytokines (IL-1β, IL-18, IL-6, IL-8 and TNF-α) were measured by qRT-PCR. The ability of PPRHs to silence *CD47* and *SIRPα* was evaluated at the mRNA level by qRT-PCR and at the protein level by Western Blot. Macrophages were co-cultured with tumor cells in the presence of PPRHs to silence CD47 and/or SIRPα. Cell viability was assessed by MTT assays.

**Results:**

THP-1 cells differentiated to macrophages with PMA showed an increase in macrophage surface markers (CD14, Mcl-1) and pro-inflammatory cytokines (IL-1β, IL-18, IL-6, IL-8 and TNF-α). PPRHs were able to decrease both CD47 expression in MCF-7 cell line and SIRPα expression in macrophages at the mRNA and protein levels. In the presence of PPRHs, MCF-7 cells were eliminated by macrophages in co-culture experiments, whereas they survived in the absence of PPRHs.

**Conclusions:**

Our data support the usage of PPRHs to diminish CD47/SIRPα interaction by decreasing the expression of both molecules thus resulting in an enhanced killing of MCF-7 cells by macrophages, which might translate into beneficial effects in cancer therapy. These results indicate that PPRHs could represent a new approach with immunotherapeutic applications.

## Background

The identification and elimination of tumor cells by the immune system on the basis of expression of tumor-specific antigens is the general concept of tumor immune surveillance which was first discussed over a century ago [1]. The critical components of the immune system involved in tumor elimination are macrophages.

Macrophage cytotoxicity in tumors can be mediated by cytokines such as tumor necrosis factor (TNF), by direct phagocytosis or combination of both [2], although primary elimination of tumor cells by macrophages occurs via phagocytosis [3]. The mechanism of tumor elimination by macrophage phagocytosis relies on distinguishing non-self molecules from self-molecules. There are several mechanisms to prevent macrophages from reacting to own tissues and there are also several mechanisms to trigger phagocytosis against tumor cells. These processes are achieved by many of the molecules and signaling pathways involved in macrophage recognition. When the specific identification of tumor cells by macrophages fails, cancer cells can evade elimination by the immune system, which has been shown as one of the hallmarks of cancer [4].

Recently, phagocytosis has been described as the result of balance between pro-phagocytic and anti-phagocytic ligands. In the last 10 years, studies have identified the large variety of pro-phagocytic molecules expressed by human tumors to induce phagocytosis and allow the immune system to eradicate them. However, a recent and promising anti-phagocytic molecule is CD47, which serves as a signal to avoid the macrophage phagocytosis when interacting its receptor Signal Regulatory Protein α (SIRPα) on macrophages [5–7].

CD47 or integrin associated protein, is an ubiquitously expressed cell surface protein in the immunoglobulin superfamily that binds many different proteins including integrins and thrombospondin-1, and it is associated with variable physiological processes including cell migration, neuronal development and T cell activation [8, 9]. CD47 consists of a highly glycosylated extracellular immunoglobulin variable (lgV) domain, a hydrophobic five transmembrane domain and an intracellular domain [10].

Poels et al. first identified CD47 as a tumor antigen on human ovarian cancer in 1986 [11]. Since then, many different human tumor types such as myeloid leukemia, non-Hodgkin’s lymphoma, bladder cancer and other solid tumors have also been found to express CD47 [8]. CD47 is widely expressed on all cell types although tumor cells have increased levels of CD47 expression compared to normal cells, which turns into a mechanism by which tumor cells can evade phagocytosis.

CD47 functions as an inhibitor of phagocytosis, since its interaction with its receptor SIRPα in macrophages leads tumor cells to be recognized as self-molecules. The interaction of CD47 with SIRPα results in the phosphorylation of immune receptor tyrosine-based inhibition motifs on SIRPα cytoplasmic tail and leads to the accumulation of myosin-IIA at the phagocytic synapse by the recruitment of Src homology phosphatase-1 and 2, which inhibit phagocytosis function [12]. Cells that display lower levels of CD47 are primed for removal, whereas cells expressing elevated levels of CD47 are resistant to clearance.

Given the importance of SIRPα-CD47 interaction for tumor growth, CD47 has been used as a validated target for cancer therapies [8]. In this study, we used Polypurine reverse Hoogsteen hairpins (PPRHs) as a gene silencing tool to decrease CD47 in tumor cells and SIRPα in PMA-differentiated THP-1 cells with the aim to decrease their interaction and to eliminate tumor cells.

PPRHs are non-modified DNA molecules formed by two antiparallel polypurine stretches. These stretches are linked by a 5-thymidine loop and the intramolecular linkage consists of reverse Hoogsteen bonds between adenine and guanine of two antiparallel stretches. When PPRHs bind their polypyrimidine target sequence by Watson-Crick bonds, they form a triplex structure, which results in the displacement of the fourth strand of the dsDNA [13, 14]. Two types of PPRHs have been described depending on the location of their target in template or coding DNA strands [15]. PPRHs bound to the template DNA strand interfere and inhibit transcription, thus decrease the mRNA and protein levels of the target gene, whereas PPRHs against the coding DNA strand are able to bind both to the coding strand of DNA and to the mRNA, since they have the same sequence and orientation [15]. PPRHs, despite performing a similar function than siRNA, do not activate the innate inflammatory response and they have demonstrated a longer half-life in mouse, human and fetal calf serum as well as in in vitro cell lines compared to siRNAs [16].

Due to the lack of purity and the limited availability of primary tissue macrophages, the THP-1 cell line is commonly used as a model of macrophages, since it resembles primary monocytes in morphology and differentiation properties [17–20]. To differentiate monocytic cell lines to macrophages, THP-1 cells are treated with PMA, which is a well-studied differentiation-inducing chemical and acts as an analog of diacyl glycerol [21]. PMA activates protein kinase C, and triggers serine/threonine kinases involved in the regulation of cellular proliferation, survival and differentiation [22, 23].

The aims of this study were: to decrease the levels of CD47 in tumor cells and SIRPα level in PMA-differentiated THP-1 cells with PPRHs and ultimately to eliminate tumor cells by macrophages by diminishing the CD47/SIRPα interaction in co-culture experiments.

## Methods

### Cell culture and PMA induced differentiation

Human acute monocytic leukemia THP-1, breast adenocarcinoma MCF-7 cell lines were used throughout the experiments.

Cell lines were grown in Ham’s F-12 medium supplemented with 7 % fetal bovine serum (FBS, both from GIBCO, Invitrogen) at 37 °C in a humidified 5 % CO2 atmosphere. Trypsinization of MCF-7 cells was performed using 0,05 % Trypsin in PBS 1× (154 mM NaCl, 3,88 mM H_2_NaPO_4_, 6,1 mM HNaPO_4_, pH 7,4). Subculture of THP-1 was performed without trypsinization depending on cell density. For differentiation, THP-1 cells were plated in 6-well dishes and induced to differentiate into macrophages using 1–3 ng/ml (**~**5 nM) phorbol 12-myristate 13-acetate (PMA) dissolved in dimetyl sulfoxide (DMSO). After PMA induction, THP-1 cells changed morphology and adhered to the culture dish. To determine macrophage differentiation, non-adherent cells were removed and mRNA levels of pro-inflammatory cytokines (IL-1β, IL-18, IL-6, IL-8 and TNF-α) and macrophage surface markers (CD14 and Mcl-1) were measured by qRT-PCR at various time points.

### Design of PPRHs

PPRHs were designed using the Triplex-Forming Oligonucleotide Target Sequence Search software (spi.mdanderson.org/tfo/, M.D. Anderson Cancer Center, Houston, TX). This software searches for polypurine sequences for the gene of interest. The output gives the location of the sequence within the gene, either the forward or the reverse strand, its exact starting point in the gene sequence and its location in the promoter, exon or intron. Only sequences with a minimum length of 20 nucleotides have been selected. After selecting proper candidates, BLAST analyses were preformed to confirm specificity of sequences and to avoid unintended targets. PPRHs were synthesized as non-modified, desalted oligodeoxynucleotides by Sigma-Aldrich (0.05 μmol scale). PPRHs were dissolved in sterile Tris-EDTA buffer (1 mM EDTA and 10 mM Tris, pH 8.0) and stored at −20 °C.

As negative controls scrambled sequences were used. Those PPRHs (Hp-Sc) do not bind to the target and have similar content in guanines than the specific PPRHs used. The sequences of the PPRHs used in this study and their abbreviations are described in Table 1.

**Table 1:** PPRHs designed against the *CD47* and *SIRPα* gene, as well as the scrambled PPRH

PPRHs against *CD47* gene	
Name	Sequence (5′-3′)
HpCD47I3-T	
HpCd47Pr-T	
PPRHs against *SIRPα* gene	
Name	Sequence (5′-3′)
HpSIRPα-Pr-C	
HpSIRPα-I7-T	
Control PPRHs	
Name	Sequence (5′-3′)
Hp-Sc	

Abbreviations are

-*Hp* PPRH hairpin

-Location within the gene sequence: Pr for promoter; I for intron and number indicates which intron

- Type of PPRH: −T for Template-PPRHs, −C for Coding-PPRHs and –Sc for scrambled PPRHs

- Letters in bold indicate polypirimidine interruptions in the sequence

### Transfection of PPRHs

Cells were plated in 6-well dishes. 100 nM PPRHs lipofected with 10 μM N-[1- (2,3-dioleoyloxy)propyl]-N,N,N-trimethylammonium methylsulfate (DOTAP; Biontex) in a volume of 200 μl of medium was incubated for 20 min at room temperature before addition of the mixture to the cells in a final volume of 1 ml.

### Anti-CD47 treatment

Cells were plated in 6-well dishes. 10 μg/mL of CD47 anti-rabbit antibody (1:20 dilution; sc-25773; Santa Cruz Biotechnology) was added to the cells in a final volume of 1 mL.

### Co-culture experiments

**Fig. 1:**
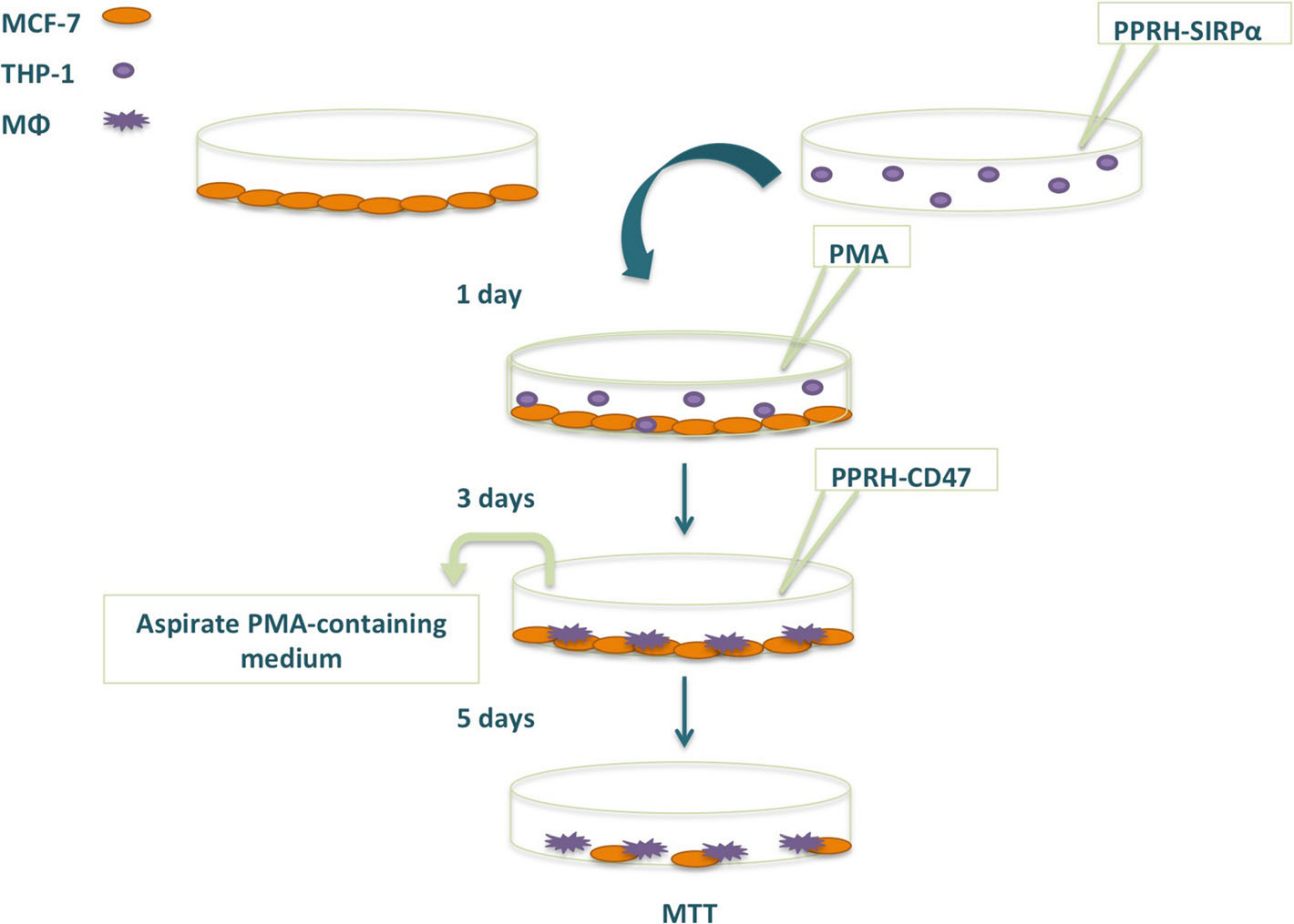
Schematic representation of co-culture experiments. MCF-7 and THP-1 cells were plated in separate dishes and THP-1 cells were immediately transfected with PPRHs against *SIRPα*. After 24 h, transfected THP-1 cells were added to MCF-7 cells and differentiated to macrophages with PMA for 3 days. After that period of time, the medium was replaced with fresh F12 medium and PPRHs against *CD47* were transfected. Cell viability (MTT) assay was assessed 5 days after the last transfection

MCF-7 (60,000) and THP-1 cells (1000) were plated in 6-well dishes separately and THP-1 cells were immediately transfected with PPRHs against *SIRPα* and the negative control, as explained in the corresponding section. After 24 h, transfected THP-1 cells were added to MCF-7 cells and treated with 3 ng/ml PMA for differentiation (Fig. 1). Three days after PMA treatment, the PMA-containing medium was aspirated and PPRHs against *CD47* and the negative control were transfected in fresh F12 medium. At the same time, antibody against *CD47* was added as positive control. Cell viability was assessed 5 days after transfection by MTT assays.

### MTT assays

Cells were plated in 6-well dishes in F12 medium in a total volume of 1 ml. Five days after transfection, 0.63 mM of 3-(4,5-dimethylthiazol-2-yl)- 2,5-diphenyltetrazolium bromide and 100 μM of sodium succinate (both from Sigma-Aldrich, Madrid, Spain) were added to the culture medium and incubated for 2.5 h at 37 °C for the reaction. After incubation, the medium was removed and the solubilization reagent (0.57 % acetic acid and 10 % sodium dodecyl sulfate in DMSO) (Sigma-Aldrich) was added. Cell viability was measured at 570 nm in a WPA S2100 Diode Array spectrophotometer. The results were expressed as the percentage of cell survival relative to the controls.

### Apoptosis assay

Apoptosis was determined by the rhodamine method: 48 h after the transfection against *CD47*, rhodamine (final concentration 5 μg/mL) (Sigma-Aldrich) was added for 30 min, the cells were collected, centrifuged at 800 g at 4 °C for 5 min, and washed once in PBS. The pellet was resuspended in 500 mL of PBS with PI (final concentration 5 μg/mL; (Sigma-Aldrich). Flow cytometry analyses were performed in a CyAn™ ADP (Beckman Coulter, Inc.) and data were analyzed using the software Summit v4.3. The percentage of Rho-negative and IP-negative cells corresponded to the apoptotic population.

### RNA extraction

Total RNA was extracted from cells using TRIzol® (Life Technologies) following the manufacturer’s specifications. RNA was quantified by measuring its absorbance at 260 nm using a NanoDrop ND-1000 spectrophotometer (Thermo Scientific).

### Reverse transcription

cDNA was synthesized by reverse transcription in a 20 μl reaction mixture containing 500 ng of total RNA, 125 ng/μl of random hexamers (Roche), 20 units of RNAse inhibitor (Lucigen), 500 μM of each dNTP (AppliChem), 2 μL of 10× buffer, and 200 units of Moloney murine leukemia virus reverse transcriptase (Lucigen). The reaction was incubated at 42 °C for 1 h.

### Real-time PCR

mRNA levels were determined by SYBR-Green Real-Time PCR in a final volume of 20 μl, containing 1× SYBR® Select Master Mix (Life Technologies), 0.25 μM of reverse and forward primers (Sigma-Aldrich), 2 or 3 μl of cDNA and H_2_O mQ up to 20 μl. PCR cycling conditions were 10 min denaturation at 95 °C, 40 cycles of 15 s at 95 °C and 1 min at 60 °C, followed by dissociation stage for 15 s at 95 °C, 20 s at 60 °C and 15 s at 95 °C.

Fold changes in gene expression were calculated using the comparative C_T_ (ΔΔC_T_) method, where C_T_ is the threshold cycle number at which fluorescence of amplified mRNA passes the threshold. GAPDH levels were used as endogenous controls. All the primers used in these experiments are detailed in Table 2.

**Table 2:** Sequences of the primers used in the qRT-PCR and the amplified product sizes

Target gene	Forward sequence (5′-3′)	Reverse sequence (5′-3′)	Product size (bp)
GAPDH	CCATGTTCGTCATGGGTGTGAACCA	GCCAGTAGAGGCAGGGATGATGTTC	251
CD14	GCAGCCGAAGAGTTCACAAG	CGCGCTCCATGGTCGATAAG	129
CD47	GAGTCTCTGTATTGCGGCGTG	GGGGTTCCTCTACAGCTTTCC	161
IL-1β	GTGGCAATGAGGATGACTTGTTC	TAGTGGTGGTCGGAGATTCGTA	124
IL-18	CCTCAGACCTTCCAGATCGC	TTCCAGGTTTTCATCATCTTCAGC	159
IL-6	CATTTGTGGTTGGGTCAGG	AGTGAGGAACAAGCCAGAGC	112
IL-8	CCACCGGAAGGAACCATCTC	TTCCTTGGGGTCCAGACAGA	279
Mcl-1	GACGAGTTGTACCGGCAGTCG	TTGATGTCCAGTTTCCGAAGC	200
SIRPα	AAATACCGCCGCTGAGAACA	TGTCCTGTGTTATTTCTCTGGCA	197
TNF-α	GCCAGAGGGCTGATTAGAG	TCAGCCTCTTCTCCTTCCTG	124

### Western blot analyses

Cells were plated in 6-well dishes and treated with 100 nM PPRHs as described in Transfection of PPRHs section. For MCF-7 cells, 3 h after transfection, total protein extracts were obtained with deoxycholate buffer (100 mM NaCl, 10 mM NaH_2_PO_4_ pH 7.4, 1 μM PMSF, 1 % triton, 0.1 % SDS and 0.5 % deoxycholic acid). Cells were washed with PBS 1× and collected by scraper in 100 μL of deoxycholate buffer. Cell debris was removed by centrifugation (13,500 × g at 4 °C for 10 min).

For THP-1 cells, cells were collected 24 h after transfection and centrifuged for 5 min at 800 g at 4 °C. Cells were resuspended in 50 μl of RIPA buffer (150 mM NaCl, 5 mM EDTA, 50 mM Tris–HCl pH 7.4 (all from Applichem, Barcelona, Spain), 1 % Igepal CA-630, 100 μg/ml PMSF and Protease inhibitor cocktail (all from Sigma-Aldrich). Cell lysate was kept on ice for 30 min, vortexing every 10 min. Cell debris was removed by centrifugation at 13,500 g at 4 °C for 10 min. The Bradford method was used to determine protein concentration using bovine serum albumin as a standard.

Whole cell extracts were resolved in 10 % SDS-polyacrylamide gels and transferred to PVDF membranes. The blocking solution was 5 % Blotto. Membranes were probed overnight at 4 °C with primary antibodies against CD47 (1:50 dilution; sc-25773, Santa Cruz Biotechnology, Heidelberg, Germany), SIRPα (1:50 dilution; sc-373896, Santa Cruz Biotechnology, Heidelberg, Germany) or GAPDH (1:100 dilution; MAB374; Chemicon International, USA). Signals were detected by secondary HRP-conjugated antibodies: anti-rabbit (1:1000 dilution; Dako, Denmark) for CD47 and anti-mouse (1:1000 dilution; sc-2005, Santa Cruz Biotechnology, Heidelberg, Germany) for GAPDH and SIRPα. Chemiluminescence was detected with ImageQuant LAS 4000 mini technology (GE Healthcare).

### Statistical analyses

All data was recorded as mean ± standard error of the mean (SE). Analyses were performed using Student’s *t* test with the software IBM SPSS Statistics v20. Significance was defined as *p* < 0.05.

## Results

### Expression of surface markers in PMA-treated THP-1 cells

**Fig. 2:**
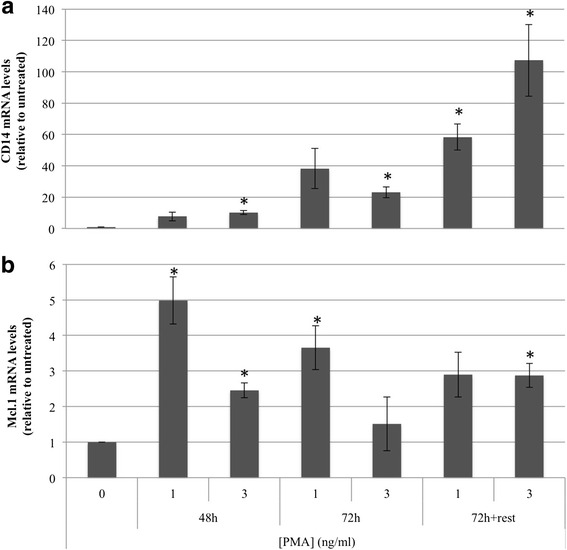
mRNA levels of macrophage surface markers. THP-1 cells (100,000) were treated with either 1 or 3 ng/ml PMA for 48 h, 72 h or 72 h + rest (72 h PMA incubation followed by 48 h resting in fresh medium). Expression of surface markers CD14 (**a**) and Mcl-1 (**b**) was determined by qRT-PCR and the results expressed relative to untreated THP-1 cells. Data represent the mean ± SE of at least three experiments (*p < 0.05, **p < 0.01, ***p < 0.005)

PMA treatment of THP-1 monocytes has been commonly used to study macrophages in vitro and different differentiation protocols including variable concentration of PMA for different time periods have been reported so far [18]. We analyzed the effect of either 1 or 3 ng/ml PMA in THP-1 cells for 48 h, 72 h or 72 h + rest (72 h followed by 48 h resting in fresh medium). Cell morphology after PMA treatment was examined under the microscope for all conditions. Cell adhesion and spreading, which are hallmarks of macrophages, were observed at all indicated time points with both 1 and 3 ng/ml PMA. CD14 [19, 20, 23–25] and Mcl-1 [26] up-regulation have been reported to be differentiation markers of macrophages upon incubation of THP-1 cells with PMA. Therefore, to determine the concentration and incubation time of PMA required to differentiate THP-1 monocytes to macrophages, mRNA levels of CD14 and Mcl-1 were determined by qRT-PCR (Fig. 2). CD14 expression was highly enhanced using 3 ng/ml PMA for 72 h + rest (Fig. 2a) and Mcl-1 expression was increased at 48 h and its elevated level maintained at 72 h + rest for both concentrations of PMA (Fig. 2b).

### PMA induced pro-inflammatory cytokines expression in a dose and time dependent manner

**Fig. 3:**
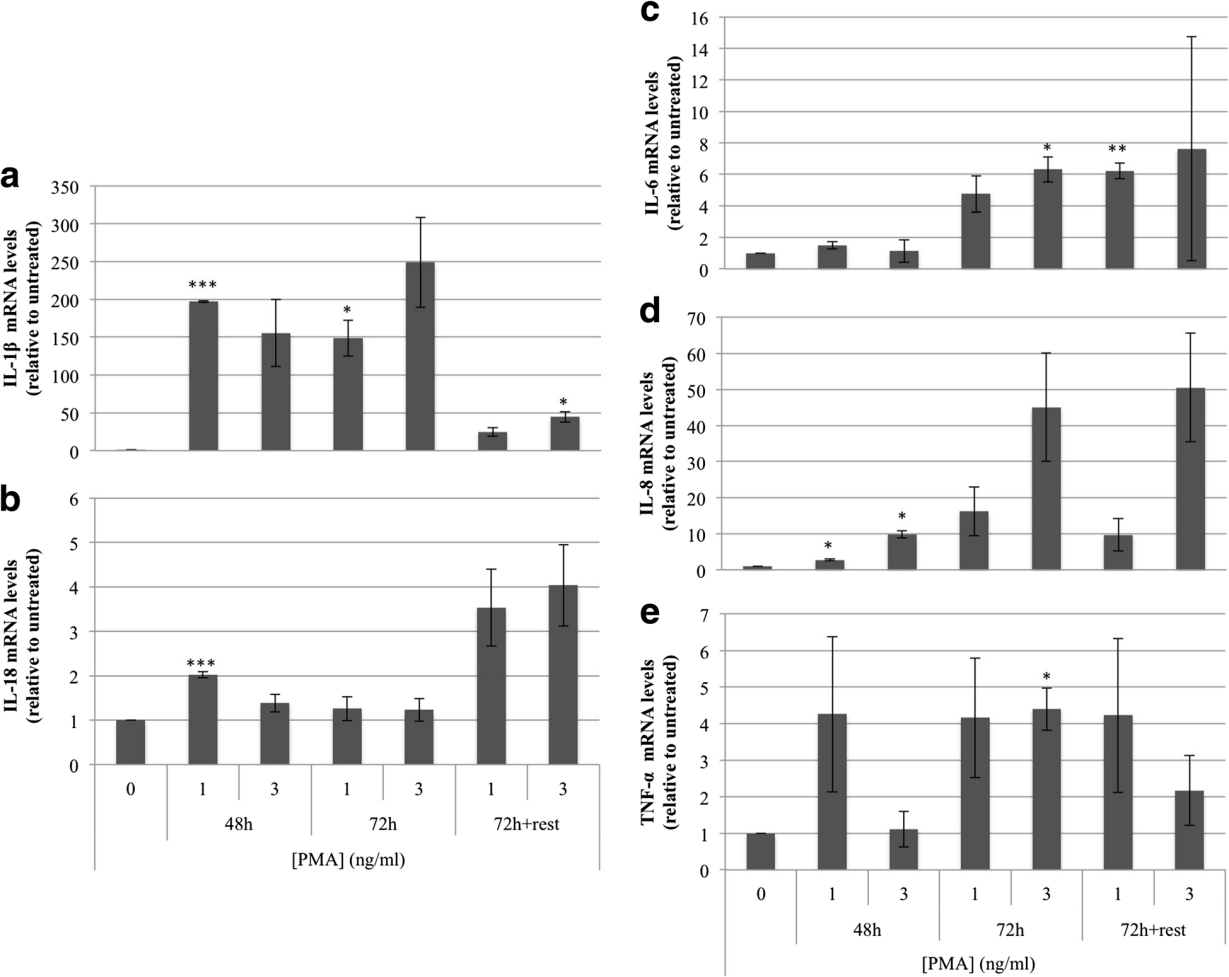
mRNA levels of pro-inflammatory cytokines. THP-1 cells (100,000) were treated with either 1 or 3 ng/ml PMA for 48 h, 72 h or 72 h + rest (72 h PMA incubation followed by 48 h resting in fresh medium). Expression of IL-1β (**a**), IL-18 (**b**), IL-6 (**c**), IL-8 (**d**), and TNF-α (**e**) was determined by qRT-PCR and the results expressed relative to untreated THP-1 cells. Data represent the mean ± SE of at least three experiments (*p < 0.05, **p < 0.01, ***p < 0.005)

Increased expression of cytokines is one of the phenotypic characteristics of differentiated macrophages [19, 20, 27]. To further investigate if THP-1 cells were able to differentiate to macrophages at 1 and 3 ng/ml PMA, mRNA expression of 5 different pro-inflammatory cytokines (IL-1β, IL-18, IL-6, IL-8, and TNF-α) was analyzed by qRT-PCR (Fig. 3). After the addition of PMA, levels of pro-inflammatory cytokines were increased dose and time dependently relative to untreated THP-1 cells. It is worth noting that the levels of cytokines produced in response to PMA are not the same depending on the culture conditions [18]. IL-1β was elevated in all conditions whereas 3 ng/ml PMA induced the highest expression when treated for 72 h (Fig. 3a). IL-18 (Fig. 3b), IL-6 (Fig. 3c) and IL-8 (Fig. 3d) stimulated at the highest level with 3 ng/ml PMA for 72 h + rest and TNF-α (Fig. 3e) increased 4.4 fold with 3 ng/ml PMA for 72 h. These results suggested that THP-1 cells could be differentiated to macrophages using 3 ng/ml PMA for 72 h and the levels of pro-inflammatory cytokines remained enhanced for the 72 h treatment followed by 48 h resting, which was also reflected in the up-regulated surface markers levels.

### Effects of PPRHs on CD47 and SIRPα levels

**Fig. 4:**
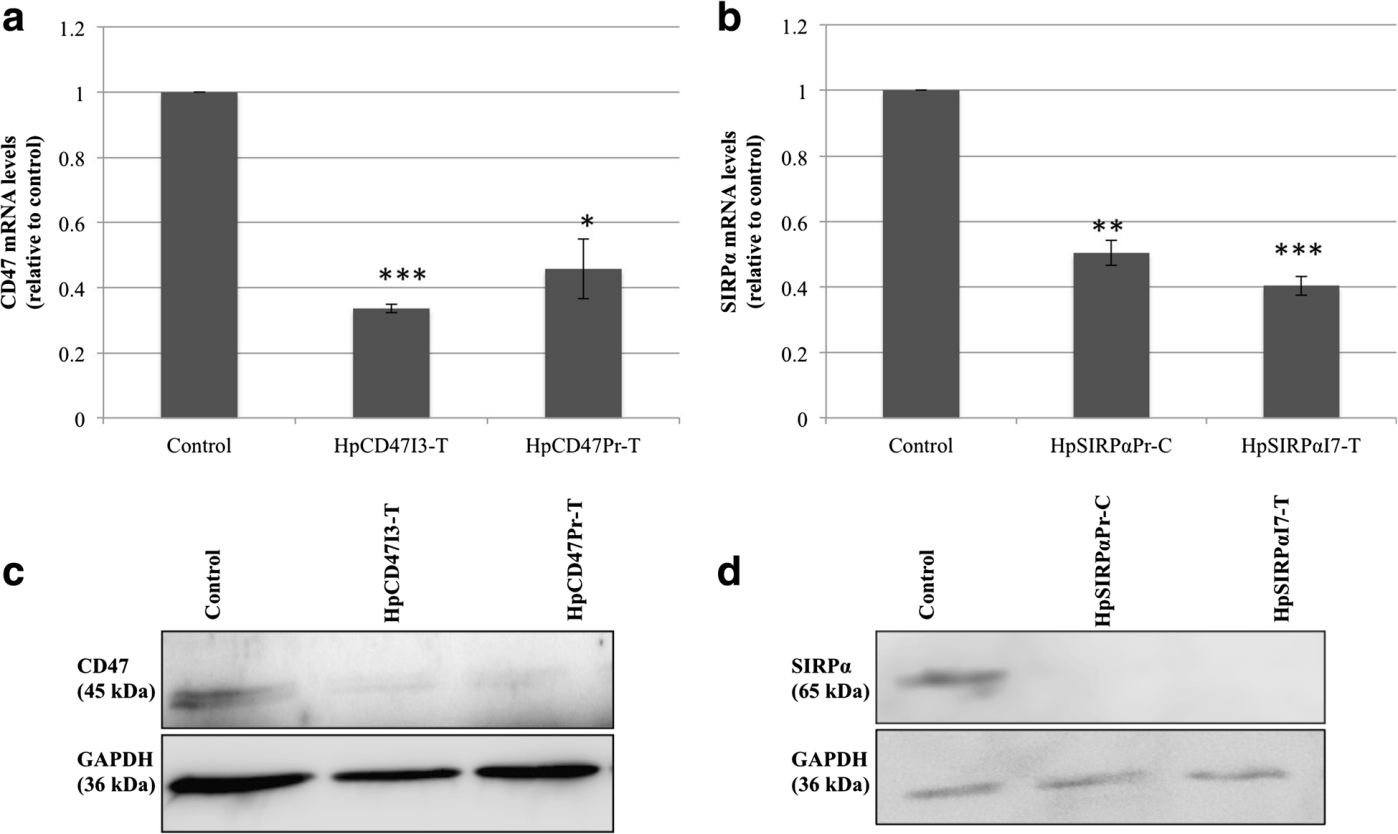
CD47 and SIRPa levels upon PPRHs transfection. **a** MCF-7 cells (60,000) were plated one day before transfection with PPRHs against CD47. RNA was extracted 24 h after transfection. mRNA levels were determined by qRT-PCR using untransfected cells as control. **b** THP-1 cells (15,000) were plated and transfected with PPRHs against SIRPα. Other conditions were as in A). Data represent the mean ± SE of at least three experiments (*p < 0.05, **p < 0.01, ***p < 0.005). **c** MCF-7 cells (60,000) were plated one day prior to transfection of PPRHs against CD47 and total protein was extracted 3 h after transfection. A representative Western blot image of CD47 protein levels upon PPRHs transfection is shown. **d** THP-1 cells (15,000) were plated and transfected with PPRHs against SIRPα and total protein was extracted 24 h after transfection. Control corresponded to untransfected cells. A representative Western blot image of SIRPα protein levels upon PPRHs transfection is shown

Two types of PPRHs against *CD47* were used in the experiments, targeting either the promoter (HpCD47Pr-T) or intron 1 (HpCD47I3-T) in the template strand of the DNA (Table 1). To explore the ability of PPRHs to silence *CD47*, CD47 mRNA levels were analyzed in MCF-7 cells either in the absence or in the presence of PPRHs. Both HpCD47I3-T and HpCD47Pr-T were able to decrease CD47 mRNA levels down to 2-fold in MCF-7 cells compared to the control (Fig. 4a). To target *SIRPα*, HpSIRPαPr-C, designed against the promoter sequence and HpSIRPαI7-T, designed against the intron 7 sequence (Table 1) were transfected in THP-1 cells. A decrease in SIRPα expression was achieved with both PPRHs (Fig. 4b). The effects of PPRHs on CD47 and SIRPα were also determined at the protein level in MCF-7 and THP-1 cells, respectively (Fig. 4c,d). Both PPRHs targeting CD47 decreased the protein level by 2.5 fold (Fig. 4c). Likewise, SIRPα protein levels were reduced by both HpSIRPαPr-C and HpSIRPαI7-T (Fig. 4d).

### Effect of PPRHs in MCF-7 cells and in Co-culture experiments

Before proceeding to co-culture experiments, we determined the cytotoxicity of the different PPRHs against either CD47 or SIRPα in MCF-7 cells. Thus, we transfected the 4 PPRHs at the same concentration of 100 nM. Whereas HpCD47I3-T and HpSIRPαPr-C caused a cytotoxicity of 48 and 28 %, respectively, HpCD47Pr-T and HpSIRPαI7-T only caused a cytotoxicity of 8 and 18 %, respectively. Given that the last two PPRHs were practically not cytotoxic in MCF-7 cells, they were selected for the co-culture experiments. The rationale for this was that we wanted to analyze the cytotoxic effect of macrophages on MCF-7 cells that had reduced CD47 levels by PPRHs, which were not cytotoxic by themselves. Also, because it was important for the co-culture experiment, we determined the lack of CD47 expression in THP-1 cells relative to MCF-7 and of SIRPα expression in MCF-7 relative to THP-1 cells (data not shown).

**Fig. 5:**
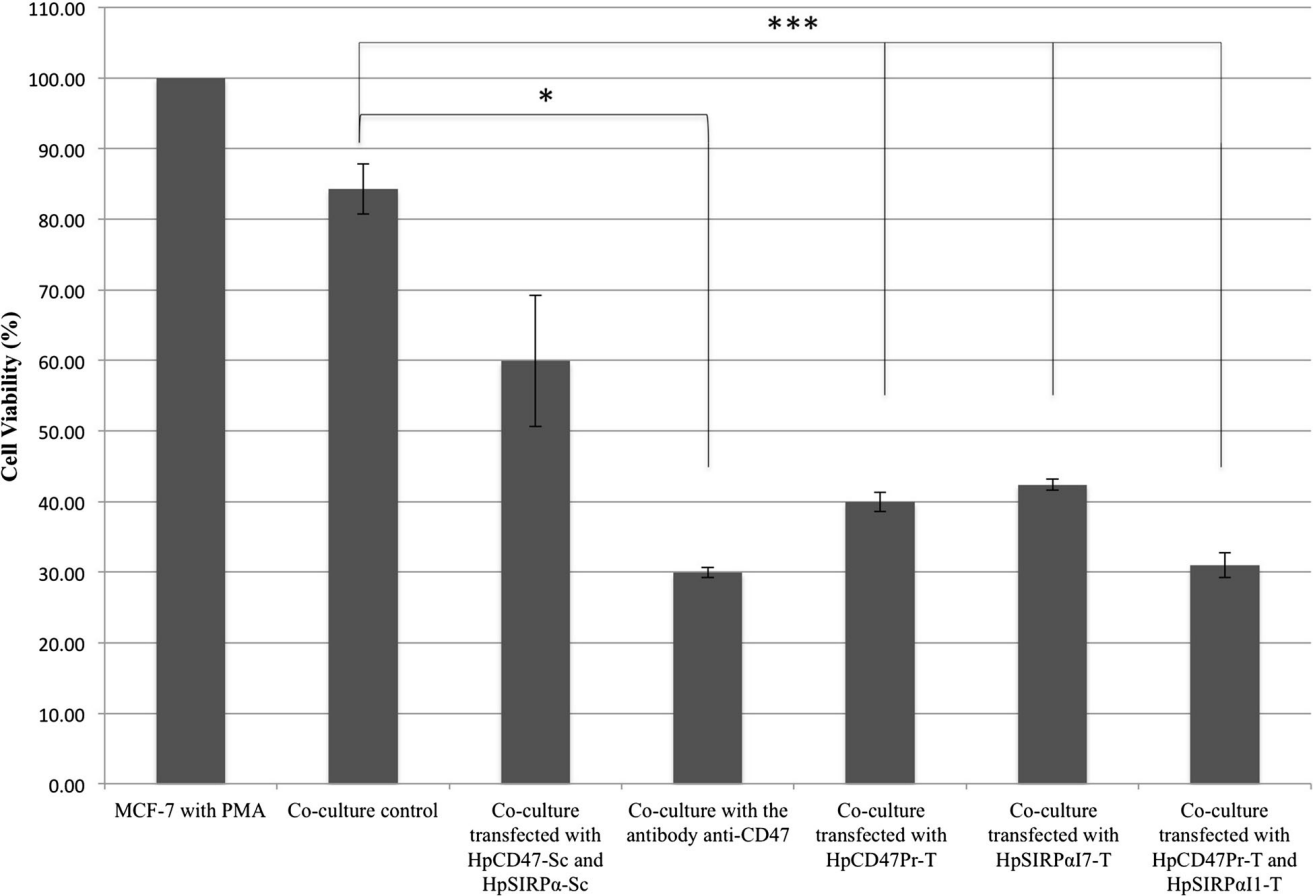
Co-culture experiments. In the co-culture experiments, either only MCF-7 cells (60,000) were transfected with HpCD47Pr-T, only THP-1 cells (1000) with HpSIRPαI7-T or both MCF-7 and THP-1 cells were transfected with the corresponding PPRH or scrambled PPRHs in controls. As positive control an antibody anti-CD47 was used. Then THP-1 cells were differentiated with 3 ng/ml PMA. The percentage of viable cells was calculated relative to the control of MCF-7 cells incubated with 3 ng/ml PMA. Data represent the mean ± SE of at least three experiments (*p < 0.05, **p < 0.01, ***p < 0.005)

Based on the patterns of pro-inflammatory cytokines and surface markers levels, 3 ng/ml of PMA was chosen for THP-1 differentiation. To decrease CD47/SIRPα interaction, CD47 and SIRPα alone or in combination were targeted by PPRHs. Scrambled PPRHs were used as negative controls and an antibody anti-CD47 as positive control to disrupt the interaction CD47/SIRPα (Fig. 5). By decreasing the level of CD47 in tumor cells, 60 % of MCF-7 cells were killed by macrophages (Fig. 5). When transfecting THP-1 cells with HpSIRPαI7-T, the decreased level of SIRPα allowed macrophages to kill 58 % of tumor cells (Fig. 5). To decrease the level of both targets in the same co-culture and to better kill tumor cells, THP-1 cells were first transfected with HpSIRPαI7-T and after differentiation, MCF-7 cells were transfected with HpCD47Pr-T in the co-culture. In these conditions, 70 % of tumor cells were eliminated. Similar results were obtained under anti-CD47 treatment. On the other hand, tumor cells escaped from macrophage killing in the absence of PPRHs and the cell viability when transfected with scrambled PPRHs was reduced by 25 % compared to that of the co-culture control (Fig. 5).

### Apoptosis assay

**Fig. 6:**
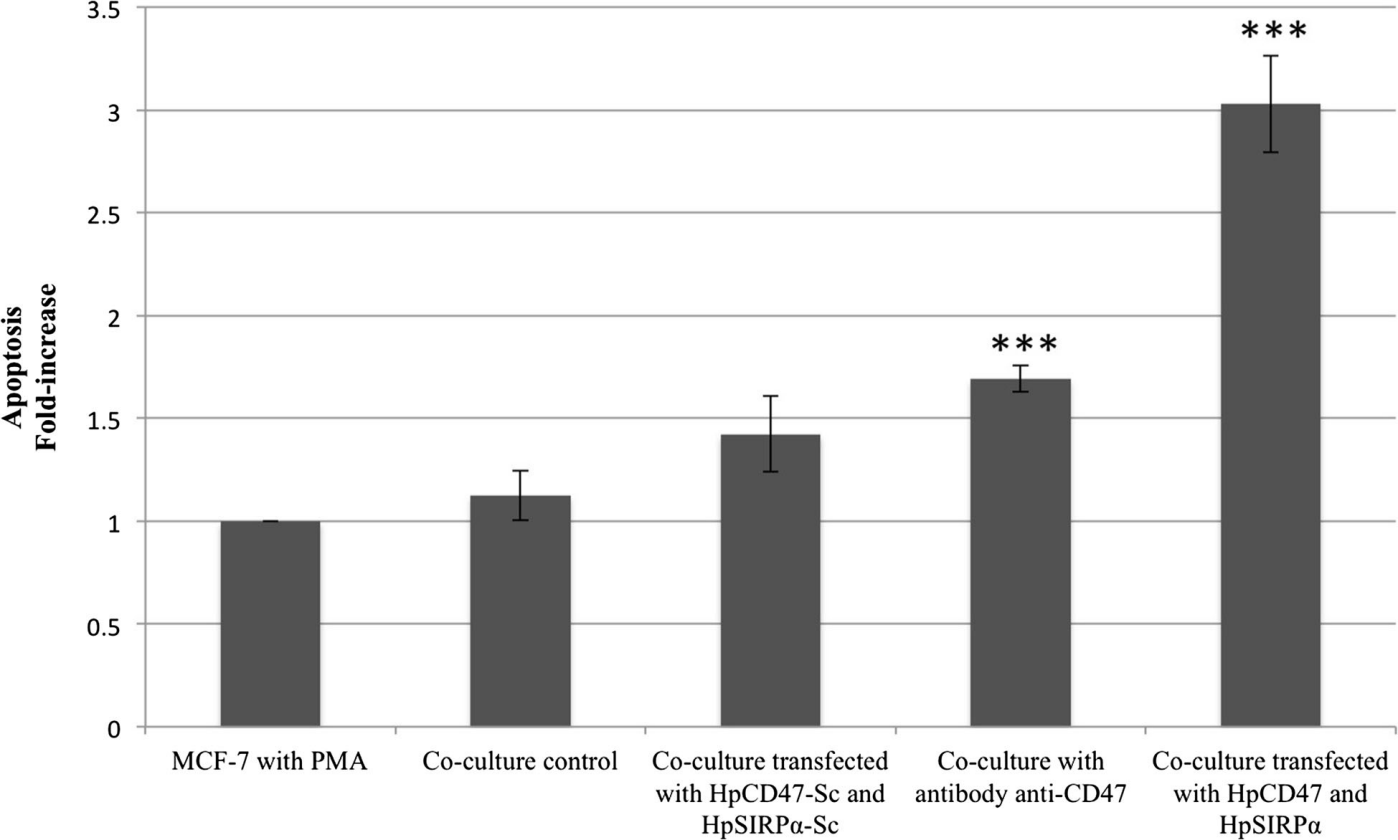
Apoptosis assay. Effect of PPRHs on apoptosis. In the co-culture experiments, either only MCF-7 cells (60,000) were transfected with HpCD47Pr-T, only THP-1 cells (1000) with HpSIRPαI7-T or both MCF-7 and THP-1 cells were transfected with the corresponding PPRH or scrambled PPRHs in controls. As positive control an antibody anti-CD47 was used. Then THP-1 cells were differentiated with 3 ng/ml PMA. 48 h after transfection, apoptosis was measured by Rhodamine method: Cells Rho123-negative and IP-negative were considered as apoptotic cells. Data represent the fold-change in apoptosis relative to MCF-7 cells treated with PMA. ^*^ *p* < 0.05

To associate the dead mechanism to the cytotoxic effect observed in the co-culture, we measured the apoptotic effect of the PPRHs at 100 nM after 48 h of incubation using the rhodamine method (Fig. 6). Co-culture transfected with HpCD47Pr-T and HpSIRPαI1-T provoked a 3-fold increase in apoptosis compared to MCF-7 cells treated with PMA, which was higher than the apoptotic effect triggered by anti-CD47 antibody (1.7-fold) used as positive control. No significant difference was detected in the co-culture control whereas the percentage of apoptotic cells in the co-culture transfected with HpCD47-Sc and HpSIRPα-Sc was twice that of the control MCF-7 cells treated with PMA.

## Discussion

In this work, we explored the usage of specific PPRHs to decrease the expression of CD47 and SIRPα to stimulate the elimination of tumor cells by macrophages, which constitutes a new approach in tumor immunotherapy.

So far, it has been shown that different concentrations and time of exposure to PMA induce THP-1 differentiation resulting in different macrophage phenotypes with altered expression of a wide range of genes at various levels. CD14 is a widely used marker to study monocyte-macrophage differentiation and its up-regulated expression is associated with macrophage phenotype [19, 20, 23–25]. The anti-apoptotic molecule Mcl-1 has also been identified as a differentiation dependent marker [22, 25]. High concentrations of PMA were previously found to induce undesirable gene up-regulation, and concentrations lower than 10 ng/ml were suggested for attain stable differentiation [18]. In the present study, we treated THP-1 cells with either 1 or 3 ng/ml PMA for incubation times commonly used in the literature (48 to 72 h), to observe the effect of different culture conditions on differentiation. Differentiation of THP-1 cells is mainly conducted with PMA treatment for 48 h or 72 h although Daigneault et al. [19] demonstrated that treating THP-1 cells with PMA followed by a period of further culture without PMA differentiates THP-1 cells to macrophages with a high capability of phagocytosis and it enhances differentiation [19]. Our data demonstrated that the highest CD14 level was reached with a PMA treatment for 72 h followed by 48 h of resting without PMA. In accordance with previous studies, we demonstrated that both CD14 and Mcl-1 levels were increased with PMA treatment in a time and dose dependent manner.

In addition to surface markers, PMA has also been reported to induce the production of pro-inflammatory cytokines in THP-1 cells [27–29]. IL-1β, IL-18, IL-6, IL-8 and TNF-α are produced by macrophages through the activation of toll-like receptor signaling, and their production is involved in the clearance of tumor cells by macrophages [30]. Our results showed the induction of several cytokines in THP-1 cells in response to PMA, depending on the duration and dose of treatment.

Cancer therapy by stimulating the patient’s immune system is one of the most promising areas of cancer research. In contrast to target the adaptive immune system, therapies have been aimed to stimulate macrophages to attack cancer. Studies have demonstrated that macrophage phagocytosis is the major mechanism when treating cancer using antibody therapies aimed to abolish the interaction between CD47/SIRPα. The efficiency of macrophage mediated tumor elimination by tumor-binding antibodies has already approved for cancer therapy [30].

As a new approach in immunotherapy, we used PPRHs, which have already been proved in gene silencing in vitro and in vivo [31]. PPRHs are more stable and show almost no immunogenicity, relative to siRNAs [16]. Since we previously described PPRHs as a gene silencing tool against different cancer targets in different human cell lines [15, 31–33], in this study we designed four different PPRHs to decrease the level of both CD47 and SIRPα and we demonstrated that all PPRHs were able to silence their target at both the mRNA and protein level. We showed that PMA-differentiated THP-1 cells eliminated tumor cells after decreasing CD47/SIRPα interaction whereas tumor cells remained unaffected in the absence of PPRHs. We also demonstrated that the mechanism responsible for observed cell death was apoptosis.

Elevated CD47 expression limits the killing of tumor cells through its interaction with SIRPα, whereas loss of CD47 triggers phagocytosis. So far, monoclonal antibodies directed against CD47 have been used to inhibit this interaction. Anti-CD47 antibodies have shown the preclinical activity in different cancers both in vitro and in animal models [6, 9, 34]. In addition to antibodies, targeting tumor CD47 using antisense strategies is another promising approach to inhibit CD47 function. Antisense morpholino oligonucleotides have been used to prevent translation of CD47 mRNA and to suppress CD47 expression in mice and miniature pigs [35, 36]. Also, antisense and siRNA strategies have been suggested to offer advantages over CD47 antibodies by avoiding many of the side effects of therapeutic CD47-antibodies such as altered blood pressure [37], hemolytic anemia and pro-thrombotic or anti-thrombotic activities [38].

Recently, Chao et al. have reported the synergic effect of antibodies against CD47 with the therapeutic cancer antibody rituximab on the phagocytosis of non-Hodgkin lymphoma by macrophages in immune-deficient mice [39]. However, it was believed that this study did not provide conclusive evidence for the role of CD47/SIRPα interaction. Furthermore, it has been shown that CD47/SIRPα and SIRPα signaling negatively regulate antibody-dependent elimination of tumor cells, which supports the idea of targeting CD47/SIRPα interaction to enhance the clinical effects of cancer therapeutic antibodies [40].

SIRPα acts to inhibit in vivo clearance of CD47-expressing host cells, including red blood cells and platelets, by macrophages [41, 42]. Therefore, direct targeting of SIRPα in immune cells, rather than CD47 in tumor cells, could be considered as an alternative approach to disrupt their interaction. A previous study showed that a novel human SIRPα-Fc fusion protein resulted in the preferential phagocytosis of acute myeloid leukemia with the blockade of CD47/SIRPα [43]. Moreover, SIRPα has been proposed as a better target due to its relatively restricted tissue expression pattern compared to CD47, which is ubiquitously expressed and binds to multiple other ligands [40].

## Conclusions

Our data support the usage of PPRHs to diminish CD47/SIRPα interaction by decreasing the expression of both molecules thus resulting in an enhanced killing of tumor cells by macrophages, which might translate into beneficial effects in cancer therapy. We believe that our results encourage the use of PPRH technology as an alternative strategy, as a new and promising immunotherapeutic approach to enhance cancer therapies.

## Abbreviations

PPRH: Polypurine reverse Hoogsteen hairpin
SIRPα: Signal regulatory protein α
PMA: Phorbol 12-myristate 13-acetate
DOTAP: N-[1- (2,3-dioleoyloxy)propyl]-N,N,N-trimethylammonium methylsulfate
